# Development of photoactive biomaterial using modified fullerene nanoparticles

**DOI:** 10.3389/fchem.2024.1432624

**Published:** 2024-11-15

**Authors:** Gabrielė Saulėnienė, Monika Kirsnyte-Snioke, Arūnas Stirkė, Vitalija Jasulaitiene, Antanas Straksys, Samuelis Dobilaitis, Wanessa C. M. A. Melo

**Affiliations:** ^1^ Department of Functional Materials and Electronics, FTMC, State Research Institute Center for Physical Sciences and Technology, Vilnius, Lithuania; ^2^ Department of Characterization of Materials Structure, FTMC, State Research Institute Center for Physical Sciences and Technology, Vilnius, Lithuania

**Keywords:** photodynamic therapy, fullerene, polylactic acid, biomaterials, nanoparticle

## Abstract

Medical device-associated biofilm infections continue to pose a significant challenge for public health. These infections arise from biofilm accumulation on the device, hampering the antimicrobial treatment. In response, significant efforts have been made to design functional polymeric devices that possess antimicrobial properties, limiting or preventing biofilm formation. However, until now none of the strategies showed a promissory effect. Thus, antimicrobial photodynamic therapy (aPDT) has been shown as a promising candidate to overcome this problem. Photosensitizers (PS) are the main key component for aPDT and fullerenes have been chosen as PS due to their good quantum yields and lifetimes spans. In this study, polylactic acid (PLA) surface was modified with fullerene (C60) and reaction was proven by XPS analysis. The biopolymer surface was characterized by AFM, SEM, and water contact angle measurements. The obtained results imply that the highest fullerene precipitation was attained when PLA was modified with ethylenediamine (EDA) before the reaction with C60, as the highest carbon increase was identified using XPS following reaction with C60. While samples’ hydrophobicity decreased after PLA modification with EDA, it increased after fullerene precipitation. Which implies that bacteria have a lower propensity to attach. Although the surface of the samples became smoother following PLA modification with EDA and reaction with 0.1% C60 precipitation, with 1% C60 precipitation the surface roughness was comparable to unmodified PLA, according to AFM and SEM analyses. Fullerene-based biopolymers could potentially be used in aPDT to make antimicrobial surfaces or medical devices.

## 1 Introduction

Medical devices have transformed healthcare over the past 50 years, saving lives by bringing new approaches to disease diagnosis, prevention, monitoring, prognosis, prediction, and treatment (Mott and Priefer, 2020). However, it has been discovered that microbial colonization and biofilm formation on these devices are linked to a risk of infections. Medical device-related infections can develop on implants, catheters, and lenses due to biofilms, which have a negative impact on quality of life and may even put lives in danger (Reid, 1999; Mack et al., 2006; Deva et al., 2013; Roberts, 2013; Khatoon et al., 2018). Indwelling medical devices can be blamed for 50%–70% of the almost two million healthcare-associated infections (HCAIs) that the Centers for Disease Control reported (VanEpps and Younger, 2016). Since biofilm is defined as surface-attached microbial cells surrounded by a self-made antimicrobial matrix (EPS), with the primary goal of protecting the self from harsh environments, immune reactions, and medicines, biofilm infections continue to be difficult to cure (Zhang et al., 2020; Sharma et al., 2019; Wu et al., 2014; Gebreyohannes et al., 2019).

Strategies for overcoming the biofilm mechanisms should focus in particular on (a) inhibiting microbial colonization and adherence to surfaces, and (b) disaggregating the biofilm matrix (Gebreyohannes et al., 2019; Kazemzadeh-Narbat et al., 2021; Mishra et al., 2023; Asma et al., 2022). Over the past 20 years, the field of antimicrobial polymers has expanded steadily, inhibiting bacteria adhesion or decreasing biofilm structure (Cattò and Cappitelli, 2019). It has been demonstrated that antimicrobial polymers can be created either (a) by impregnating them with antimicrobial agents (antibiotics, disinfectants, etc.) or (b) by inserting functionalities with antimicrobial activity into the polymer’s backbone or side chains (Francolini et al., 2015). In the first method, the antimicrobial ingredient is carried by the polymer, which, once it is released, performs its effect. To create intrinsically antimicrobial polymers (biocidal polymers), bactericidal functions are added to the polymer in the second method (Riga et al., 2017). Biocidal polymers kill bacteria as they come into touch with the surface rather than releasing antimicrobial chemicals (Chen et al., 2017). They don’t theoretically exhaust their activity because they don’t emit antibacterial chemicals (Chen et al., 2017). Despite encouraging findings, not all recently developed antibiofilm surfaces have gone through the crucial process of validating their antibiofilm performance (Roy et al., 2018). Likewise, many experimental polymeric antibiofilm surfaces reported in the literature have never been applied in actual settings. This could be explained by the fact that antimicrobial polymers are typically specialized for a single microbial species, which could lead to intrinsic mutagenesis and enhance host cell harm (Riga et al., 2017).

Polymers could be treated with photosensitizers (PS) to perform antimicrobial photodynamic treatment (aPDT) as a way to get around the revealed information. The aPDT is a potential treatment that offers benefits such a wide therapeutic window, diverse targets (no microbial resistance development), and selective death of microbial cells with little harm to the human tissues (Mylona et al., 2020; Bispo et al., 2021). Additionally, aPDT has been demonstrated to be an effective therapy in numerous laboratory animal models of bacterial and fungal infections (Sellera et al., 2019; Hu et al., 2018; Kashef and Hamblin, 2022; Pérez-Laguna et al., 2019; Fila et al., 2016), in clinical veterinary practice (Hengzhuang et al., 2016; Valandro et al., 2021; Grego et al., 2017), and in clinical trials for chronic wounds and dentistry in humans (Fila et al., 2016; Al-Shammery et al., 2019; Simonetti et al., 2011). The concentration of PS, its physicochemical characteristics, the irradiation time (which depends on the light source), and the morphology of the microorganisms are all important factors that affect aPDT efficiency (Alves et al., 2014). A chemically pure drug that is specifically absorbed by the target tissue, has a high triplet quantum yield to encourage the production of ROS, and does not exhibit dark toxicity (activated only by radiation) are all characteristics of the ideal PS (St Denis and Hamblin, 2015; MacRobert et al., 1989).

Fullerene is a unique and versatile material that has been widely studied for its potential applications in various fields, including medicine, chemistry, and materials science. One of the most intriguing properties of fullerene is its ability to be activated by a wide range of light wavelengths, including UVA, blue, green, and white light (Sharma et al., 2011; Hamblin, 2018). This property has led to its exploration as a photosensitizer (PS) in photodynamic therapy (PDT), a treatment modality that uses light-activated compounds to destroy cancer cells and other undesirable cells (Nanashima and Nagayasu, 2015; de Melo et al., 2013; Chen et al., 2012; Prylutska et al., 2014; Fernandes et al., 2022; Grebinyk et al., 2018).

The advantages of fullerenes over other PS classes, include (a) versatile functionalization (Hu et al., 2018; Lu et al., 2022), (b) light-harvesting antenna (Hou et al., 2022; Agazzi et al., 2019), (c) ability to undergo Type 1 and Type 2 photochemistry (Hu et al., 2018; Hamblin, 2018; Mroz et al., 2007), (d) electron transfer’s ability to result in oxygen-independent photokilling (Hu et al., 2018; Hamblin, 2018; Hou et al., 2022; Agazzi et al., 2019; Yin et al., 2015), (e) the ability to self-assemble into supramolecular fullerosomes (Hu et al., 2018; Verma et al., 2005; Albert and Hsu, 2016), (f) the ability to self-assemble into theranostic nanoparticles (Hu et al., 2018; Verma et al., 2005; Albert and Hsu, 2016), (g), components of theranostic (Hu et al., 2018; Kawasaki et al., 2022).

The study of modifying polylactic acid (PLA) with fullerene is a novel concept that has not been previously explored. The combination of these two materials has the potential to create new properties that could be utilized in various applications, particularly in the field of medicine. One such application is photodynamic therapy (PDT), a treatment modality that utilizes a photosensitizer, light and oxygen to induce cell death in malignant cells. The incorporation of fullerene into PLA could enhance the photo-sensitizing properties of the polymer, making it more efficient in PDT. Additionally, the modified PLA-fullerene polymer could also be used to create antimicrobial materials for medical devices. Fullerene has been shown to possess antimicrobial properties, and incorporating it into PLA could enhance the antimicrobial properties of the polymer, making it more suitable for use in medical applications where preventing bacterial contamination is crucial. Overall, the study of modifying PLA with fullerene is of great importance as it has the potential to improve the properties of the polymer, and open up new opportunities for applications in the field of medicine.

The aim of this work was to synthesize a photoactive fullerene-coated biopolymer that could be used in aPDT. Thus, for future perspectives evaluate the application of this biomaterial as a medical device. Furthermore, this study aims to make a significant contribution to the field of photodynamic therapy by developing a novel and potentially more effective treatment option for patients.

## 2 Materials and methods

### 2.1 Fullerene-based biopolymer synthesis

#### 2.1.1 General description of PLA formation

15 mL of dichloroethane (DCE) were poured to a 25 mL conical tube along with 2.813 g of PLA (Biopolymer 6202D, IngeoTM) (VWR Chemicals). The mixture was heated at 60°C for 2 h with constant stirring to achieve complete dissolution. The resulting solution was then cooled to room temperature. Polyethylene terephthalate (PET) (3M Italia S.p.A) was then cut into round disks with a diameter of about 2 cm, and processed in low-pressure plasma using a Zepto plasma system (Diener electronic, Plasma—Surface—Technology) instrument at 100 power for 10 min in an O^2^ environment to increase PET surface hydrophilicity. This was done to prepare the surface for PLA surface formation.

By adding 2 mL of PLA solution to PET, spin-coating it at 10,000 rpm for 20 s, and then accelerating to 13,000 rpm for 60 s, an even layer of PLA was created. The samples are placed in an oven at 60°C for 30 min to allow the solvent to completely evaporate.

#### 2.1.2 PLA modifications to bind with fullerene C60

**FIGURE 1:**
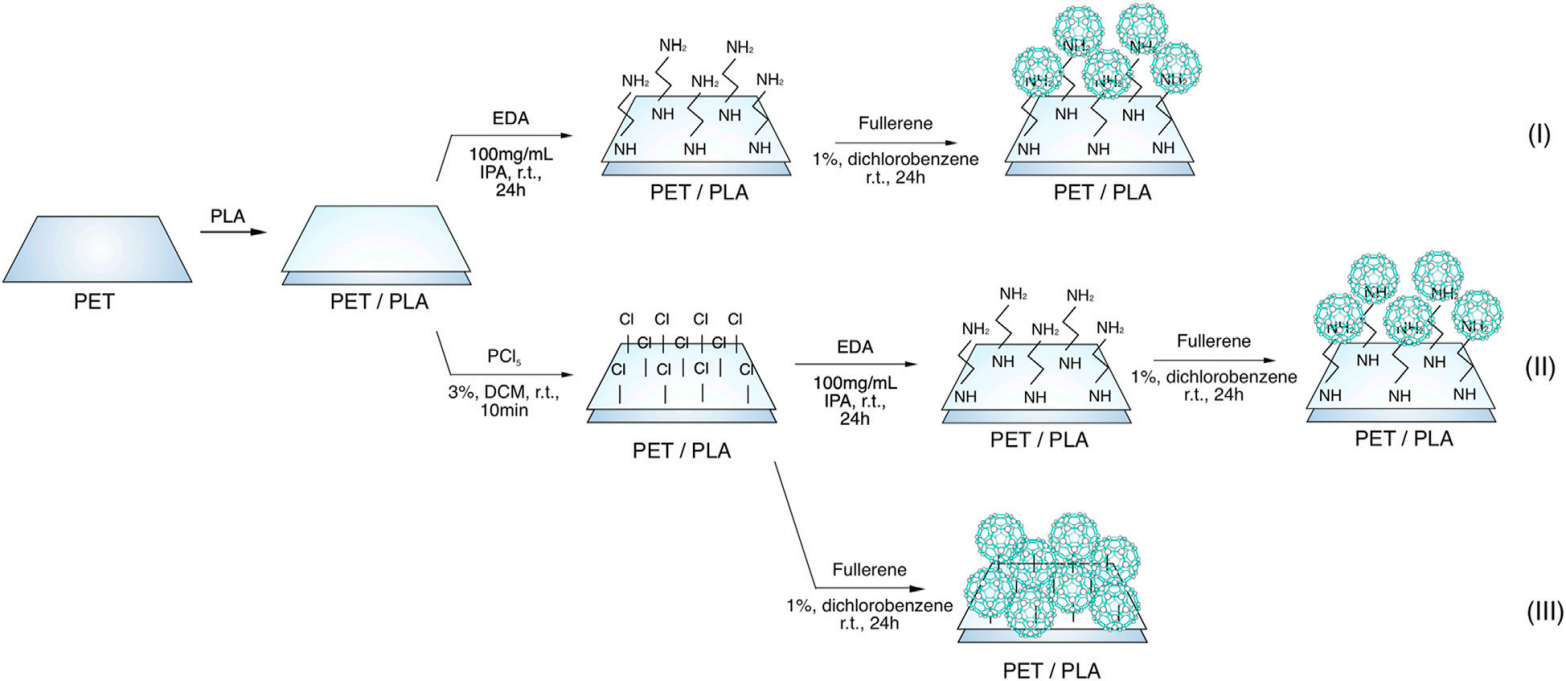
General scheme of Fullerene-based biopolymers synthesis.

To bind with C60, polylactic acid was modified in three different ways, as described below (Figure 1).

I) Polylactate samples were incubated in an isopropanol (IPA) solution containing 100 mg/mL of the ethylenediamine (EDA) (Alfa Aesar) for 24 h at room-temperature. Before reacting with C60, the samples were washed with isopropanol (VWR Chemicals) and dried at room-temperature. To immobilize C60 on the surface, previously modified PLA was immersed in 1% dichlorobenzene (Sigma-Aldrich) solution of C60 (Sigma-Aldrich) at 25°C for 24 h. The samples were then dried overnight at room-temperature after being washed with dichlorobenzene.
II) Polylactate samples were immersed in 3% phosphorous pentachloride (PCl_5_) in dichloromethane (DCM) (VWR Chemicals) for 10 min, followed by a DCM wash and room-temperature drying. The ethylenediamine and C60 reaction is conducted under the same conditions as those mentioned for route I.
III) Polylactate samples were immersed in 3% PCl_5_ in dichloromethane for 10 min, followed by a dichloromethane wash and room-temperature drying. Then, using 0.1% and 1% C60 in dichlorobenzene, the reaction is carried out under the same conditions as those mentioned in route I.

### 2.2 Chemical and structural analysis

A variety of analytical techniques were used to study the chemical composition and structure of the samples acquired in the experiment. These techniques included X-ray photoelectron spectroscopy (XPS), atomic force microscopy (AFM), scanning electron microscopy (SEM), and water contact angle (WCA). Each of these techniques provides unique information about the samples and can be used in combination to gain a more understanding of the samples.

#### 2.2.1 XPS measurements

XPS measurements were used to verify data regarding the elemental chemical states and surface composition of the samples using a Kratos AXIS Supra X-ray photoelectron spectrometer with monochromatic Al Kα radiation (hυ = 1486.6 eV) operated at 225W with a base pressure of 1.7 × 10^−9^ mbar in the analysis chamber. Wide scan spectra were acquired at 160 eV pass energy and 1.0 eV step size. The data were quantified by measuring peak areas and using Scofield sensitivity factors modified for the instrument geometry and the inelastic mean free path energy dependency. The intensity/energy response of the instrument was determined following acquisition of spectra from copper, silver and gold. High energy resolution spectra at 10 eV pass energy and 0.1 eV step size were acquired for chemical state determination, and peak fitting was employed to determine the position and intensity of overlapping photoelectron peaks. Charge referencing was relative to Au4f_7/2_ line at 84.0 eV (Briggs, 1990). All data processing was carried out using Avantage processing software v.5.9922 (ThermoScientific, United Kingdom). The area of the XPS analysis was 700 × 300 μm.

#### 2.2.2 Atomic force microscopy

AFM (BioscopeII/Catalyst, Bruker, United States) was applied for the surface visualization and characterization of PLA and its alterations with EDA and C60. A silicon nitride probe (DNP, Bruker) coated with a gold reflecting coating and having a tip radius of 0.55 μm and a nominal resonance frequency of 18 kHz was used to capture AFM pictures. Images with a 250 nm contrast and a 10 μm × 10 µm size were recorded.

#### 2.2.3 Scanning electron microscopy

The samples were covered with a thin layer of a few nanometers of chromium to improve their resolution and contrast when observed under a Helios NanoLab 650 scanning electron microscope. The Helios NanoLab 650 scanning electron microscope is a high-resolution and high-performance SEM that is equipped with a high-resolution detector and high-performance electron optics. It allows for high-resolution imaging of samples at different magnifications, which is essential for studying samples at the nanometer scale. The 20 kV accelerating voltage that was used in this experiment is suitable for imaging samples with a thin chromium coating. It allows for the imaging of surface features with high resolution and minimal beam-induced damage to the samples.

#### 2.2.4 Water contact angle

The surface wettability was ascertained with the goniometer (Easy Drop, Kruss) by measuring the contact angle of a droplet on a sample surface of route I. Droplets water, PBS and LB medium were used of room temperature. A small (5 µL) drop of liquid was placed on the sample’s surface in order to measure the angle between the drop and the surface in the lateral projection.

#### 2.2.5 Absorbance

The absorbance was characterized using UV−vis spectrophotometry. UV–vis spectrophotometer (UV-1800, Shimadzu) was used to measure the absorbance spectra at wavelength interval of 200–800 nm.

### 2.3 Determination of ROS generation in fullerene-based biopolymer

The generation of ROS from the fullerene-based biopolymer samples was performed following the protocol outlined in the publication (Černý et al., 2010), with slight modifications. Blue LEDs with a predominant wavelength range of 450–500 nm and a light intensity of 50 W/m^2^ were used for sample irradiation. ROS were generated from the PLA modified with fullerene C60 samples using a blue light source designed and manufactured in FTMC. The ROS test was conducted using dimethylformamide (DMF) (Sigma-Aldrich) as the solvent, and the stability of 9,10-diphenylanthracene (DPA) (Fluorochem) under these conditions was confirmed. A calibration curve was generated, yielding a correlation coefficient of *R* ^2^ = 0.997. The optical density of the final volume did not exceed 1.5.

Firstly, fullerene-based biopolymer films with varying concentrations of fullerene C60 were weighed and rinsed with dry DMF to remove any residual impurities. The films were then transferred into vials containing 4 mL of DMF and 0.2 mL DPA solution in DMF, ensuring the optical density remained below 1.5. This setup was used to assess the amount of ROS generated by the fullerene-based films.

Secondly, the vials containing the fullerene-based PLA samples were placed in a holder and irradiated with blue light for 2 h. A vial without the sample served as a control. Following irradiation, the vials were removed, and the absorbance of the solution was measured at 395 nm using a UV-vis spectrophotometer (Halo RB-10, Dynamica). In this condition the quantum yield of ROS production was calculated following equation:

$$ROS\;\left(\text{mol}\right)=initial\;DPA\;\left(\text{mol}\right)-measurement\;DPA\;\left(\text{mol}\right)$$ (1)

## 3 Results

### 3.1 Synthesis fullerene-based biopolymers

Due to the absence of reactive sidechain groups, PLA is chemically inert, making modification challenging (Casalini et al., 2019). Therefore, its surface was modified was modified in three different ways, as described below. XPS analysis was used to carefully analyze the PLA modifications and reactions fullerene C60 as shown in Figure 1.

#### 3.1.1 Synthesis route I

**FIGURE 2:**
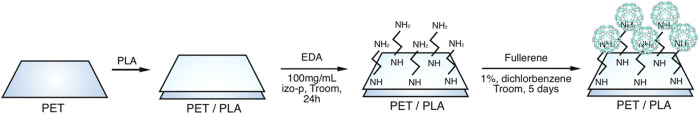
Synthesis of Fullerene-based biopolymers route I.

The ethylenediamine/isopropanol solution was applied to PLA for 24 h at a concentration of 100 mg/mL. The samples were then dried for an hour at room temperature after being washed with isopropanol, and then C60 was applied (Figure 2). Dichlorobenzene was used to dissolve C60 before it was added to the samples. Room temperature was used for the process. All samples were washed and allowed to dry at room temperature for a further 24 h.

An observation was made in the N1 spectrum where a new peak at a wavelength of 398.6 eV was detected following reactions with EDA. This peak indicates an increase in the amount of atomic nitrogen present, from a previous level of 0.4% to a new level of 6.7%. This increase in atomic nitrogen suggests that a new bond has been formed as a result of the reaction with EDA.

**FIGURE 3:**
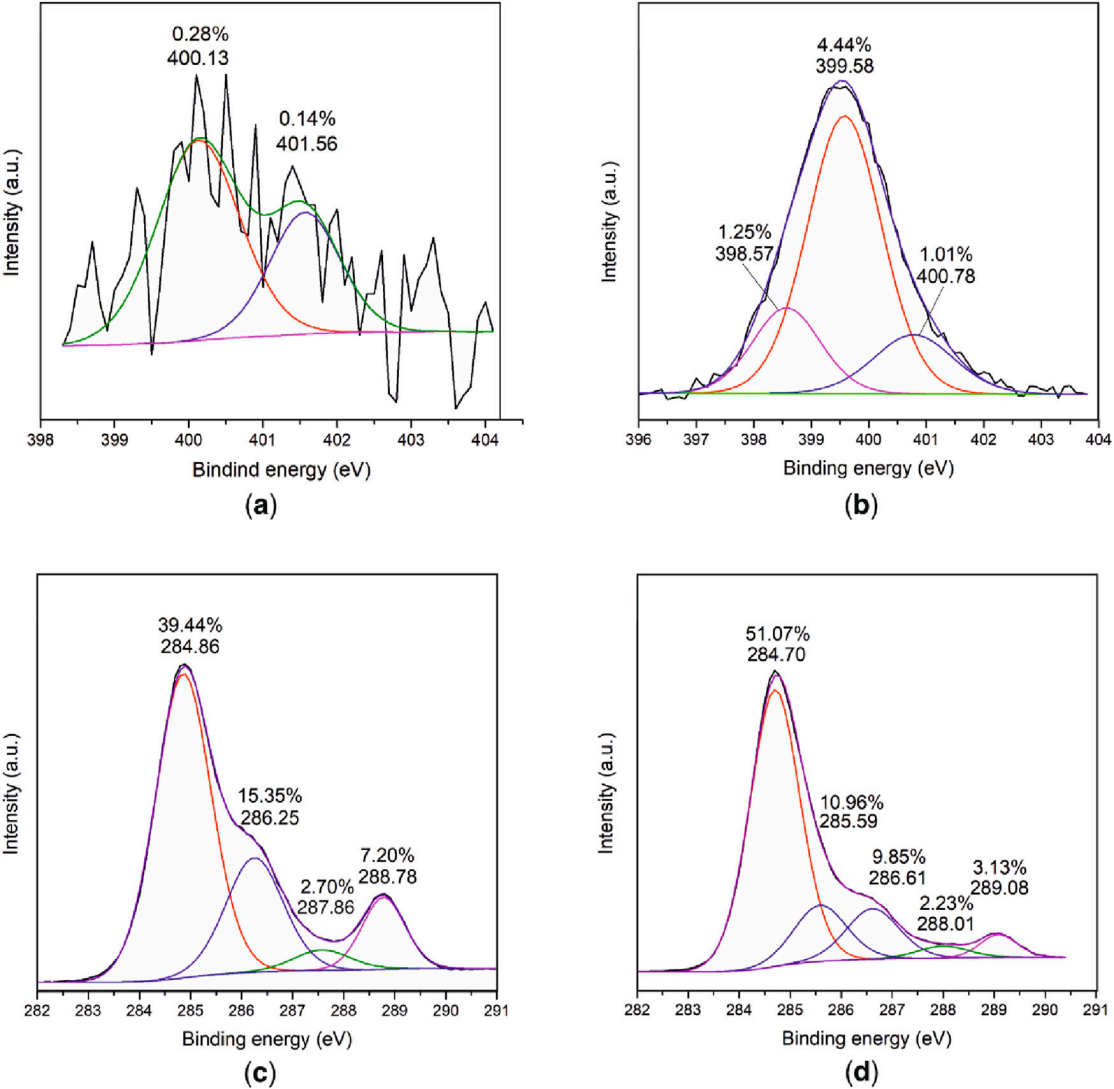
XPS spectra analysis of PLA modification with EDA and reaction with fullerene C60. N1 spectra: **(A)** PLA, **(B)** PLA modified with EDA. C1 spectra: **(C)** PLA modified with EDA, **(D)** PLA modified with EDA after reaction with 1% C60.

An examination of the carbon C1 spectrum revealed a new peak at a wavelength of 289 eV following a reaction with C60, as shown in Figure 3D. The observed peak indicates the formation of new π-π interactions, originating from the C60 molecule. This confirms the attachment of C60 to the surface. Additionally, the analysis revealed an increase in atomic carbon content from 64.7% to 88.1%, further supporting the presence of C60 on the surface.

#### 3.1.2 Synthesis route II

Phosphorous pentachloride has previously been used to activate polylactic acid, and this has been followed by chemical conjugation with the surface-modifying agent EDA that contains an amine (-NH_2_) group (Ning Luo et al., 2004; Rahul, 2009; Park et al., 2018).

**FIGURE 4:**
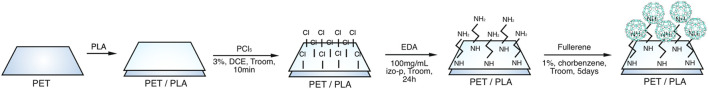
Synthesis of Fullerene-based biopolymers route II.

After being exposed to a 10 min treatment with a 3% solution of PCl_5_ in dichloromethane, the PLA were washed with dichloromethane and dried (Figure 4). The following step involves performing the ethylenediamine and fullerene reaction under the same conditions as those mentioned for route I.

X-ray photoelectron spectroscopy (XPS) measurements revealed that the atomic nitrogen content increased from 0.4% to 7% after the reaction with pentylene chloride and ethylenediamine. Further analysis of the N1 spectrum revealed the presence of a new peak at a wavelength of 398.6 eV following the reaction with EDA, which is indicative of the formation of a new bond. This new peak also corresponds with an increase in the atomic nitrogen concentration from 0.4% to 6.9% (Figures 3A, B).

Furthermore, after the reaction with C60, the atomic carbon content was found to have increased from 64.7% to 76.3%. Additionally, a new peak at 289.3 eV was observed in the carbon C1 spectra (Figure 3C), which confirms the presence of C60-derived bonds, as reported in reference (Sahoo and Patnaik, 2003).

#### 3.1.3 Synthesis route III

**FIGURE 5:**
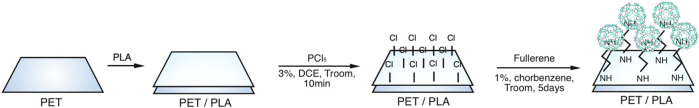
Synthesis of Fullerene-based biopolymers route III.

Since direct reactivity with fullerene is conceivable after PLA activation with PCl_5_ (Ning Luo et al., 2004), PLA was immersed in 3% phosphorus pentachloride in dichloromethane for 10 min, then rinsed with dichloromethane and dried (Figure 5). The reaction is then carried out using 1% C60 in dichlorobenzene under the same conditions outlined for route I.

An XPS analysis prior to the reaction with phosphorus pentachloride (PCl_5_) did not detect any atomic chlorine. However, after the reaction with PCl_5_, a measurable quantity of atomic chlorine was detected, at 13.7%. Also, after the interaction with C60, the XPS analysis showed that atomic carbon content increased from 61.8% to 84.6% (Figure 3).

Additionally, a new peak was observed at 288.9 eV in the carbon C1 spectrum (Figures 3C, D), which is indicative of the formation of π-π bonds, as reported in reference (Sahoo and Patnaik, 2003). This observation confirms that C60 precipitation on the modified PLA surface. Table 1 provides an overview of different routes used in the experiment and it was found that the highest precipitation was achieved during route I.

**TABLE 1:** Change of atomic carbon content after reaction with fullerene.

Route	Change of atomic carbon content after reaction with fullerene, %
I	23.4
II	6.9
III	22.8

### 3.2 AFM analysis

**FIGURE 6:**
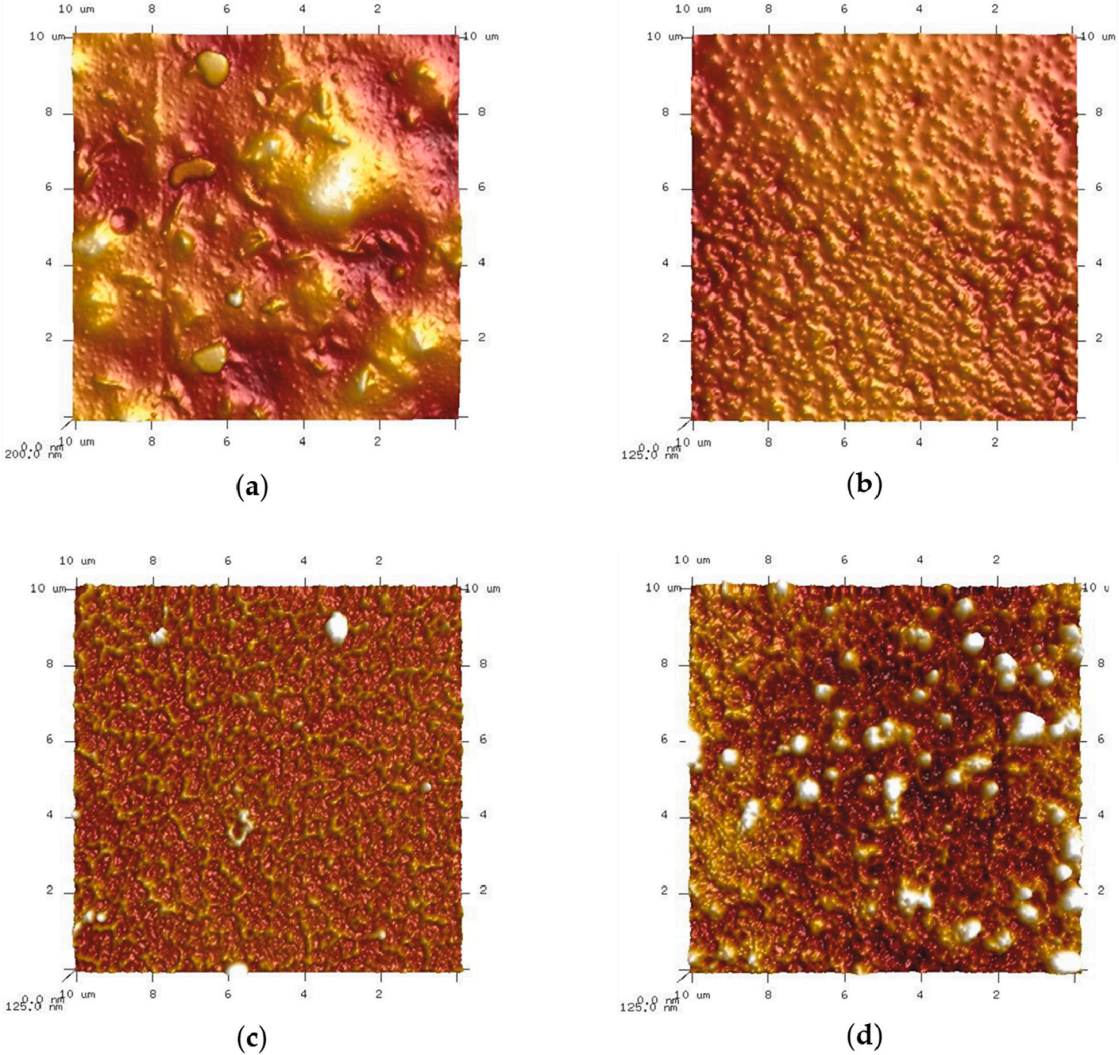
AFM topography 3D view: **(A)** PLA; **(B)** PLA modified with EDA; **(C)** PLA modified with EDA after reaction with 0.1% C60; **(D)** PLA modified with EDA after reaction with 1% C60.

Roughness and imperfections on a surface have been found to enhance cell adhesion (Ning Luo et al., 2004). Atomic force microscopy (AFM) was used to analyze the surface topography and roughness. The only samples scanned were those from route I, which had the greatest change in atomic carbon content after reacting with fullerene C60. Images with a 250 nm contrast and a 10 m × 10 m size were obtained (Figure 6).

The surfaces of pure PLA, PLA modified with EDA and PLA modified with EDA after reactions with 0.1% and 1% C60 were scanned using an atomic force microscope to assess 3D surface smoothness. Distinct differences in surface topography were observed among the samples, highlighting the effects of EDA modification and C60 concentration on PLA surface characteristics. The surface of plain PLA was found to have wide, high protrusions and tiny, dot-like “peaks” as irregularities, which is similar to the structure obtained by L. Lei when PS was covered by PLA and dissolved in chloroform (Ning Luo et al., 2004). However, when PLA was modified with EDA, only tiny, dot-like “peaks” were detected in the surface analysis.

Following reactions with 0.1% and 1% C60, the surface roughness of the samples increased, with more prominent and wider protrusions observed, particularly in the sample treated with 1% C60, consistent with findings from Prylutskyy et al. (2017). These observations align with those reported for C60 fullerene nanoparticles deposited on mica surfaces, which exhibit significant roughness, dot-like peaks, and broad protrusions.

To evaluate the formed irregularities, the arithmetic average roughness value (Ra) was calculated for the extensions and shown in Table 2. It has been observed, that surface roughness has a tendency to diminish following EDA treatment, and increase following reactions with 0.1% and 1% C60.

**TABLE 2:** Ra roughness values of PLA, PLA modified with EDA, and PLA modified with EDA after reaction with 0.1% and 1% C60.

Material	Ra roughness, μm
PLA	0.038
PLA modified with EDA	0.005
PLA modified with EDA after reaction with 0.1% C60	0.015
PLA modified with EDA after reaction with 1% C60	0.031

### 3.3 SEM

**FIGURE 7:**
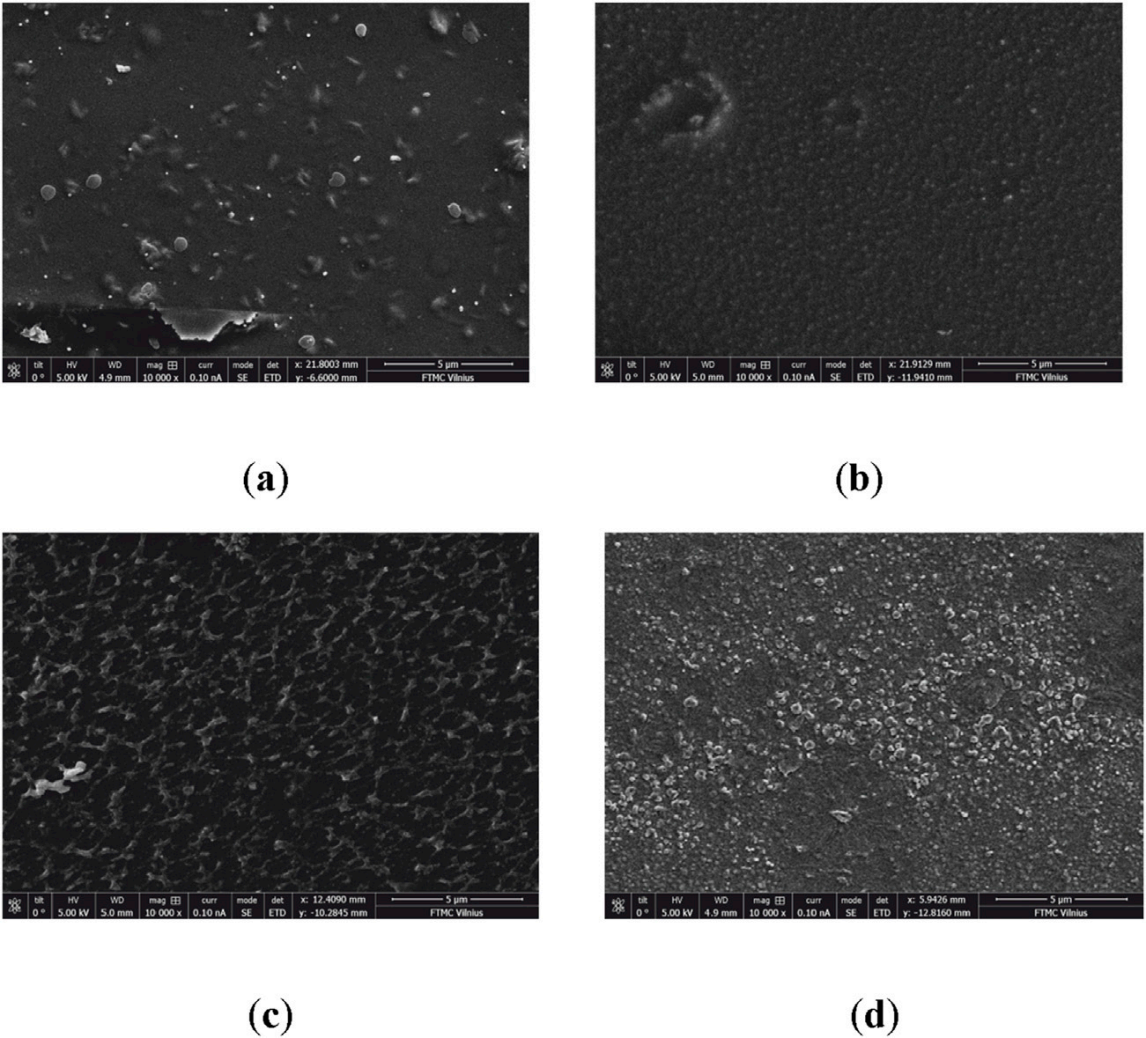
SEM topography 3D view: **(A)** PLA; **(B)** PLA modified with EDA; **(C)** PLA modified with EDA after reaction with 0.1% C60; **(D)** PLA modified with EDA after reaction with 1% C60.

The surface topography of PLA, PLA modified with EDA, and PLA modified with EDA after reactions with 0.1% and 1% C60 was evaluated using SEM as a complement to AFM surface analysis (Figure 7).

PLA has surface irregularities, while PLA modified with EDA has fewer but larger protrusions, which are comparable to those we could see in the AFM analysis. However, after reacting with C60, the topography of the surface changes and it exhibits taller peaks and different patterns. The SEM surface depiction is comparable to the AFM surface visualization because of the higher and wider protrusions, particularly following reaction with 1% C60.

**FIGURE 8:**
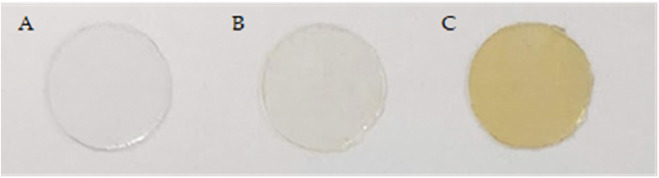
**(A)** PLA, **(B)** PLA modified with 0.1% fullerene, **(C)** PLA modified with 1% C60.

Figure 8 illustrates the yellowish tint that resulted from the reaction with C60, this color intensity is directly proportional to the percentage of C60 used in the reaction. The yellow coloration is thought to be due to the presence of C60 on the surface and the interaction between C60 and the surface.

### 3.4 Water contact angle measurements

**FIGURE 9:**
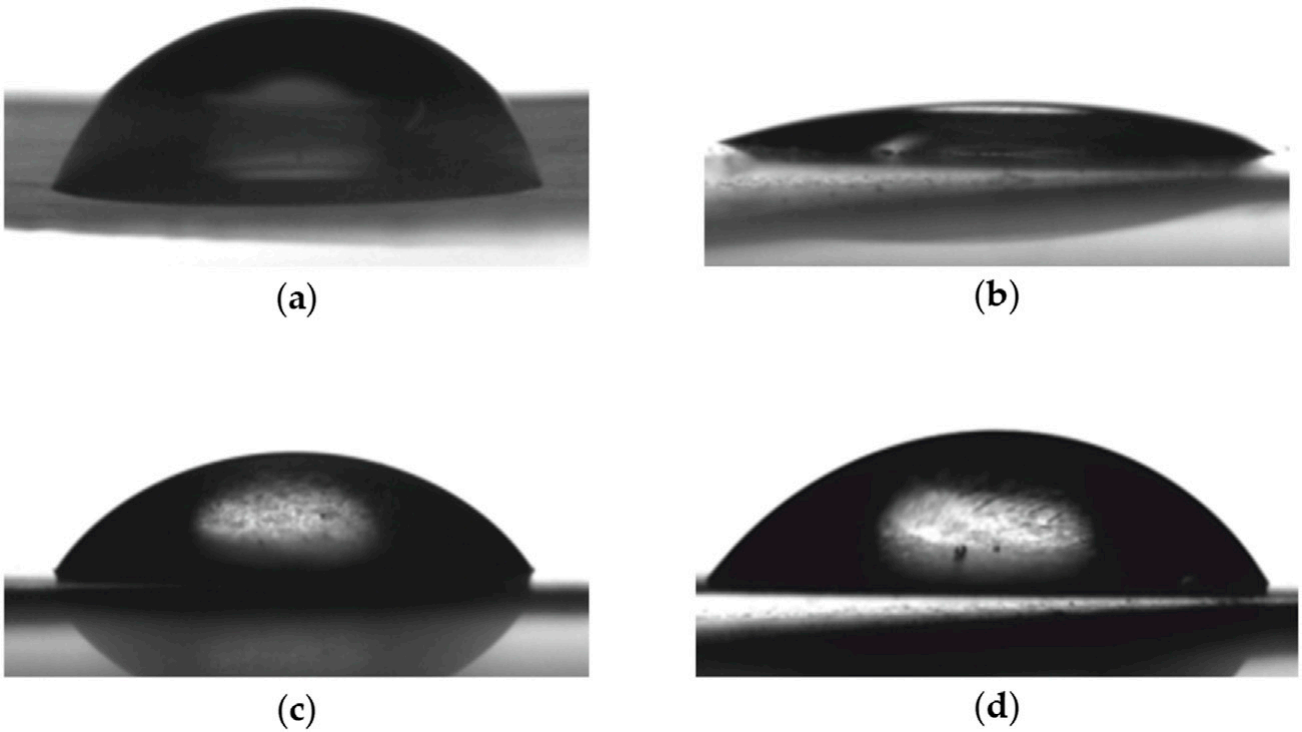
Water contact angle images: **(A)** PLA; **(B)** PLA modified with EDA; **(C)** PLA modified with EDA after reaction with 0.1% C60; **(D)** PLA modified with EDA after reaction with 1% C60.

To assess surface hydrophobicity, the water contact angle (WCA) was measured. An increase in WCA indicates a decrease in hydrophilicity. In order to determine the angle between the drop and the surface, drops of room temperature water, PBS, and LB medium were placed on the surfaces of PLA, PLA modified with EDA, and PLA modified with EDA after reactions with 0.1% and 1% C60. WCA analysis indicates apparent variations across samples, which are displayed in Figure 9; Table 3 respectively.

**TABLE 3:** Comparisons of WCA of different surfaces.

Sample	Contact angle (degrees)		
	H_2_O	PBS	LB
PLA	75	66	71
PLA modifies with EDA	18	15	18
PLA modifies with EDA after reaction with 0.1% C60	64	58	61
PLA modifies with EDA after reaction with 1% C60	70	66	68

Experimental results demonstrated that the water contact angle (WCA) of the samples significantly decreased following modification with EDA, dropping from 75° to 18°. However, subsequent reaction with C60 resulted in an increase in WCA, ranging from 64° to 70°. Additionally, the experiment examined the effects of other solutions, including phosphate-buffered saline (PBS) and Luria-Bertani (LB) medium, both commonly used in biofilm production. The lowest WCA was observed between EDA-modified PLA and PBS (15°), whereas the highest WCA was recorded between unmodified PLA and water (75°). These findings indicate that the contact angle was highest with water and lowest with PBS among the tested solutions. The EDA modification rendered the surface more hydrophilic, whereas the subsequent reaction with C60 increased hydrophobicity.

### 3.5 Absorbance measurements

**FIGURE 10:**
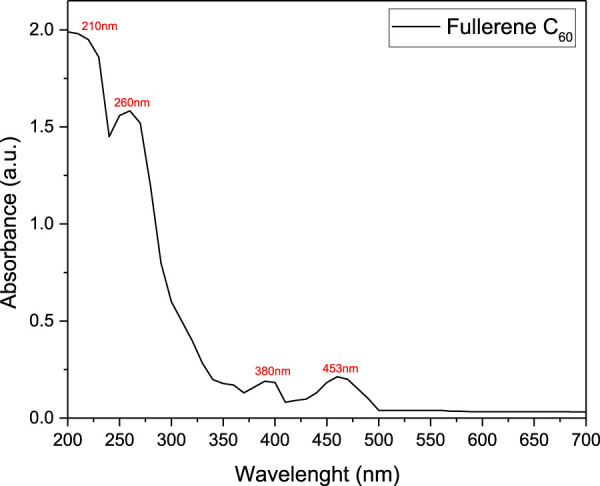
Absorbance spectra for synthetized PLA modified with EDA and C60.

The UV-Vis spectrum of fullerene C60 when combined with PLA and EDA presents distinct peaks in both the UVB and UVA regions (Figure 10). The UV-Vis spectrum displays prominent peaks at **210 nm** and **260 nm** in the **UVB** range. These peaks correspond to π-π* transitions within the C60 molecular structure and are characteristic of the molecule’s conjugated electron system. The peaks here are slightly shifted and somewhat intensified due to interactions with the PLA matrix, which can stabilize certain electronic states of C60.

In the **UVA** region, two key absorption peaks are observed at **380 nm** and **453 nm**. These peaks are indicative of n-π* transitions, which are sensitive to changes in the molecular environment. The absorption at **453 nm**, in particular, is notably higher in the composite than in fullerene C60 dissolved in a usual organic solvent like DMSO (Saraswati et al., 2019), likely due to the influence of EDA. EDA can interact with C60 via weak chemical bonding or electrostatic interactions, altering the distribution of electron density and leading to shifts in the UV-Vis absorption profile.

Thus, combining C60 with PLA provides a stable matrix that minimally affects the absorption characteristics of C60, helping to preserve its UV-visible spectrum without significantly altering peak positions or intensities. EDA may interact with C60 through amine bonding, potentially affecting the stability or intensity of certain transitions, though the primary UV peaks (210, 260, 380, and 453 nm) remain largely intact.

### 3.6 ROS determination in fullerene-based samples

In the study of ROS determination in PLA modified with fullerene C60, ROS generation was quantified using a reaction with 9,10-diphenylanthracene (DPA) as an indicator. Fullerenes mobilized onto the surface of the PLA biopolymer generated ROS, which reacted with DPA to produce 9,10-diphenyl-9,10-dihydro-9,10-epidioxyanthracene. The concentration of ROS produced was calculated by the difference between the initial and measured amounts of DPA according to the Section 2.3 (Equation 1). Experimental results indicated that the concentration of ROS generated per gram of the modified biopolymer increased with fullerene content. Specifically, PLA modified with 1%, 5%, and 10% fullerene C60 produced ROS at levels of 1.81E-05, 4.10E-05, and 1.77E-04 mol/g, respectively. This increase in ROS concentration with higher fullerene loading demonstrates the potential of fullerene-modified PLA films for enhanced antibacterial activity through ROS-mediated mechanisms.

## 4 Discussion

As previously discussed, polylactate (PLA) is known to be chemically inert, making it challenging to modify (Casalini et al., 2019). Therefore, to bind with fullerene C60, surface of polylactate was modified. As it was previously discovered, PLA modification with EDA induce aminolysis that results in breakage of ester bonds and formation of amide bonds, -NH_2_ and -OH groups which could further react to C60 (Yu et al., 2018; Zhu et al., 2013; Zhu et al., 2012). PLA aminolysis is possible due to amine group which acts as a nucleophile to attack PLA at the electron deficient site -C=O (Mittal et al., 2010). The formation of reactive NH_2_ groups on the surface of the PLA, as confirmed by XPS analysis which shows an increase in nitrogen content and the appearance of a new peak in the N1 spectra. It is worth noting that this process of PLA modification has been previously studied by other researchers, with similar results. For example, Xu et al. (2011) conducted a similar reaction using aliphatic diamine and found that after a 12-h reaction, a new peak was seen in the N1 spectra, indicating the formation of reactive NH_2_ groups on the surface of the PLA. This further confirms the effectiveness of the PLA aminolysis technique for modifying the surface of the polymer and allowing for the attachment of C60. Once the surface of the PLA has been modified, it can then be reacted with C60, which is known to react with diamines in accordance with a coupling reaction mechanism (Uluğ et al., 1997; Kenneth Sauer and Puglisi, 2001; Olah, 1992; Miller, 2006; Javahery et al., 1993; Eklund and RaoHancké, 2022). This reaction is confirmed by XPS results, which show an increase in atomic carbon content and the formation of a new peak at 289 eV in the carbon C1 spectrum. This new peak is believed to be the result of the formation of π-π bonds originating from fullerene C60 (Dong et al., 2018), indicating that it has successfully attached to the surface of the PLA.

In this study it was considered alternative sequences of chemical reactions when attempting to improve the yield of fullerene C60 precipitation on PLA surfaces. It is well known that surface activation can improve the adhesion of certain molecules to a substrate, but in this case, it did not result in higher C60 precipitation. Instead, the study suggests that modifying the PLA with EDA before reacting with C60 (route I) results in the highest C60 precipitation, as seen by the highest carbon increase detected by XPS analysis. It is important to note that the study may have limitations and the results may not be generalizable to all systems, and further research is needed to fully understand the underlying mechanism of the observed phenomena. Additionally, it would be valuable to explore other potential routes for modifying PLA surfaces that could lead to improved C60 precipitation. Overall, this study provides new insights into the optimization of C60 precipitation on PLA surfaces, and suggests that the sequence of chemical reactions may play a crucial role in determining the success of such processes.

AFM and SEM analysis indicate that the modification of PLA with EDA and subsequent reaction with 0.1% C60 results in a smoother surface compared to unmodified PLA. However, increasing the C60 concentration to 1% leads to surface roughness levels comparable to that of unmodified PLA. These findings are particularly relevant for applications such as medical devices and antibacterial coatings, where surface topography plays a critical role in cell adhesion (Ning Luo et al., 2004). Increased surface roughness can promote bacterial or cellular attachment, potentially affecting the material’s antibacterial efficacy and biocompatibility.

The hydrophobicity of the samples is also an important factor to consider as it can affect the attachment of microorganisms to the surface. The results of this study show that after PLA modification with EDA, the samples become less hydrophobic, which suggests that microorganisms are more likely to attach. However, C60 precipitation increases the hydrophobicity of the samples, which may lead to decreased attachment of microorganisms. This is an important consideration when using this material for applications where the attachment of microorganisms needs to be minimized.

Also, in this study, we demonstrate a clear correlation between fullerene C60 concentration and ROS production in PLA modified with fullerene biopolymer. As the concentration of fullerene on the PLA surface increases from 1% to 10%, the ROS production rises significantly, with ROS concentrations of 1.81E-05 mol/g and 1.77E-04 mol/g, respectively. This notable increase in ROS generation suggests that the C60 content directly influences ROS production, consistent with fullerene’s well-documented role as a photosensitizer (Hamblin, 2018). When exposed to light, fullerenes absorb energy and transfer it to surrounding oxygen molecules, generating ROS, including singlet oxygen (^1^O₂) and superoxide anions (O₂⁻). This mechanism aligns with previous studies on fullerene-based materials, showing similar ROS generation patterns and their subsequent antibacterial effects (Lahir, 2017). The increase in ROS concentration with fullerene content enhances the material’s oxidative potential, which is crucial for its intended antimicrobial applications. Furthermore, these results suggest that adjusting the C60 concentration in PLA, makes it possible to control ROS production levels, enabling the design of materials with tunable antibacterial properties.

Photobleaching, however, can occur with fullerene C60 under prolonged light exposure, where the molecule undergoes gradual oxidative degradation, leading to a reduction in its absorbance and photosensitizing efficiency. This degradation is primarily driven by the generation of ROS when C60 absorbs energy from light, causing ROS such as singlet oxygen and superoxide anions to form and react with the fullerene molecule itself. In high-oxygen environments, these ROS can degrade the carbon cage structure of C60, diminishing its ability to absorb light and produce further ROS (Lissi et al., 1993). Despite this tendency, incorporating C60 into a PLA matrix has been shown to stabilize the molecule against photobleaching. The PLA matrix acts as a protective barrier that reduces oxygen exposure to C60, thereby lowering the rate of ROS-induced self-degradation (Mao et al., 2018). Additionally, the PLA encapsulation minimizes molecular motion, which further decreases the likelihood of oxidative breakdown. As a result, C60 embedded in PLA maintains its photosensitizing ability over a longer duration, allowing for more sustained ROS production in applications where antimicrobial or photocatalytic effects are desired (Lienkamp et al., 2009).

Our results revealed that this modified PLA modified with EDA and C60 exhibited heightened photoactivity in the UVB region. Although, the material demonstrated some degree of photoactivity in the visible spectrum, suggesting that the synthetized material could potentially be used in aPDT. A potential application for photoactive fullerene-based biopolymers in photodynamic treatment is also suggested by the fact that fullerene has an extended π-conjugation, absorbs visible light, has a high triplet yield, and can produce reactive oxygen species when illuminated (Hamblin, 2018). In comparison to most other PS chemical structures used in PDT applications, fullerenes are more versatile since they can perform Type 1, Type 2, and electron transfer photochemical reactions, the balance of which may be customized by making the relevant modifications (Hamblin, 2018). Along with significant UV absorption, fullerenes mostly absorb visible light at blue and green visible wavelengths (Hamblin, 2018). It is safe to apply visible light, therefore materials made or covered with fullerene-modified polylactic acid could be used in contact with human skin and make medical devices such as catheters, patches or coatings.

In conclusion, the study of modifying PLA with fullerene C60 is of great importance as it has the potential to improve the properties of the polymer, and open up new opportunities for applications in various fields, particularly in the field of medicine. Further research in this area could lead to the development of new materials with unique properties and improved performance, which could have a significant impact on society and industry. The potential applications of photoactive fullerene-based biopolymers in photodynamic treatment are promising and warrant further research. The versatility and biocompatibility of these materials, along with the ability to perform multiple types of photochemical reactions, make them an attractive option for the development of new medical devices and therapeutics.

## Data Availability

The original contributions presented in the study are included in the article/supplementary material, further inquiries can be directed to the corresponding author.
